# Impact of a Very Low-Calorie Ketogenic Diet (VLCKD) on Changes in Handgrip Strength in Women with Obesity

**DOI:** 10.3390/nu14194213

**Published:** 2022-10-10

**Authors:** Luigi Barrea, Giulia de Alteriis, Giovanna Muscogiuri, Claudia Vetrani, Ludovica Verde, Elisabetta Camajani, Sara Aprano, Annamaria Colao, Silvia Savastano

**Affiliations:** 1 Dipartimento di Scienze Umanistiche, Università Telematica Pegaso, Via Porzio, Centro Direzionale, Isola F2, 80143 Napoli, Italy; 2 Centro Italiano per la Cura e il Benessere del Paziente con Obesità (C.I.B.O), Unit of Endocrinology, Dipartimento di Medicina Clinica e Chirurgia, Federico II University Medical School of Naples, Via Sergio Pansini 5, 80131 Napoli, Italy; 3 Unit of Endocrinology, Dipartimento di Medicina Clinica e Chirurgia, Federico II University Medical School of Naples, Via Sergio Pansini 5, 80131 Napoli, Italy; 4 Cattedra Unesco “Educazione Alla Salute e Allo Sviluppo Sostenibile”, University Federico II, 80131 Napoli, Italy; 5 PhD Program in Endocrinological Sciences, University of Rome “La Sapienza”, 00161 Rome, Italy; 6 Department of Human Sciences and Promotion of the Quality of Life, San Raffaele Roma Open University, 00166 Rome, Italy

**Keywords:** ketogenic diet, VLCKD, inflammation, handgrip strength, muscle mass, fat mass, obesity, nutritionist

## Abstract

The preservation of muscle mass, which is positively associated with muscle strength, has been included among the benefits of ketogenic diets due to the synergistic effects exerted by the reduction in visceral adipose tissue and obesity-related pro-inflammatory status. The handgrip strength (HGS) test is widely used as a single indicator to represent overall muscle strength. The possible association of changes in HGS in patients with obesity during the consumption of a very low-calorie ketogenic diet (VLCKD) has not yet been investigated. The aim of this prospective study was to assess the efficacy of VLCKD on promoting changes in HGS and high-sensitivity C-reactive protein (hs-CRP) levels, as a serological marker of obesity-related, low-grade inflammation, in a population of women with obesity after 45 days of active phase of the VLCKD. This pilot, uncontrolled, single-center, open-label clinical trial examined 247 Caucasian women, aged 18–51 years (body mass index, BMI: 30.0–50.9 kg/m^2^) who were consecutively enrolled following 45 days of active phase the VLCKD. Anthropometric measures and physical activity were evaluated. Muscle strength was measured by HGS using a grip strength dynamometer. Body composition was evaluated using a bioelectrical impedance analysis (BIA) phase-sensitive system. hs-CRP levels were determined by nephelometric assay. Adherence to the VLCKD, ketosis status, and physical activity were checked weekly by phone call. At day 45, BMI, fat mass (FM), and hs-CRP levels were significantly decreased (∆-7.5 ± 3.1%, ∆-15.6 ± 9.0%, and ∆-39.9 ± 44.6%; respectively; *p* < 0.001 for all three parameters), while HGS had increased (∆+17.4 ± 13.2%; *p* < 0.001). After adjusting for ∆BMI, ∆waist circumference, ∆hs-CRP levels, and physical activity, the correlation among changes in ∆HGS (kg), ∆FM (kg), and ∆ fat free mass (FFM) (kg) remained statistically significant (r = −0.331, and r = 0.362, respectively; *p* < 0.001). Interestingly, the correlation between ∆HGS with ∆FM (r = −0.288, *p* < 0.001) and ∆FFM (r = 0.395, *p* < 0.001) were also independent of the percentage of weight loss. We are the first to report that, along with a significant reduction in body weight and an overall improvement in body composition and inflammatory status, the muscle strength evaluated by the HGS test increased in a population of women with obesity after 45 days of the active phase of the VLCKD, also following adjustment for common confounding variables.

## 1. Introduction

Recently, very low-calorie ketogenic diets (VLCKD) have been regaining interest in the panorama of nutritional strategies to manage obesity and related disorders commonly linked to low-grade inflammation and dysfunctional visceral adipose tissue [1,2,3,4]. The European Association for the Study of Obesity (EASO) guidelines highlight that VLCKD may be used only as part of a lifestyle intervention for weight loss under the supervision of a clinical nutritionist [5]. In addition, VLCKD should be limited to specific patients and for a short time frame. In 2019, the consensus statement from the Italian Society of Endocrinology (SIE) strongly recommended VLCKDs in several clinical conditions, but only after excluding the absolute contraindications [6] Table 1.

According to the EASO guidelines, the protocol is characterized by three stages: active, reeducation, and maintenance [5]. During the active phase of the VLCKD, no more than 800 calories (kcal) per day are allowed, carbohydrates (<50 g per day from vegetables) and lipids (10 g of olive oil per day) are reduced, and 0.8–1.5 g/kg ideal weight of protein are provided [4,7]. The side effects of the VLCKD are generally mild, easily managed, and transient [4,8]. The main goal of ketogenic diets (KDs) is to cause nutritional ketosis by replacing glucose as a source of energy in most tissues over time with ketone bodies [9]. The ability to switch between glycolysis and ketosis promotes a variety of favorable changes in metabolic parameters and body composition [10]. In particular, ketone bodies increase satiety and decrease hormone-dependent hunger, which cooperate to improve patient compliance [5]. In addition, ketone bodies also exert antioxidant and anti-inflammatory actions [2]. The short-term beneficial effects of the VLCKD on serological markers of inflammation were observed in a medium-sized case series of patients with obesity (both genders) by Monda et al. [11]. In this context, the preservation of muscle mass has been included among the benefits of KDs, due to the synergistic effects exerted by the reduction in visceral adipose tissue and obesity-related pro-inflammatory status, and the modulation of gut microbiota [12,13,14]. In addition, Merra et al. [15] demonstrated that, after 3 weeks of dietary intervention, KD was effective in reducing body weight without inducing muscle mass loss, thus preventing the risk of sarcopenia. However, in KD patients, the preservation of muscle mass, a factor known to be involved in glucose metabolism, is still questionable [5]. Several studies have reported that muscle mass is positively associated with muscle strength [16,17,18,19]. In particular, the handgrip strength (HGS) test is widely used as a single indicator to represent overall muscle strength [20]. Nevertheless, it is well known that the HGS test varies according to age, sex, and race, and that certain comorbidities, including obesity, have significant impacts on muscle strength due to a reduced ability to engage in regular physical activity or an increase in intramyocellular fat [16]. In addition, the relationship between subclinical systemic inflammation, muscle strength, and/or muscle mass in adults has been recently revised in a systematic review and meta-analysis [21]. In the complex scenario of the beneficial effects of the VLCKD, beyond its well-documented weight-loss effectiveness, the possible association of changes in HGS and low-grade inflammation has not yet been investigated in patients with obesity.

The aim of this prospective study was to assess the efficacy of the VLCKD in promoting changes in HGS and high-sensitivity C-reactive protein (hs-CRP) levels, as a serological marker of obesity-related, low-grade inflammation, in a population of women with obesity after 45 days of the active phase of the VLCKD.

## 2. Materials and Methods

### 2.1. Design and Setting

This open-label, pilot, single-center, prospective clinical study has been investigator-initiated to evaluate the efficacy of the VLCKD in promoting changes in HGS in a population of women with obesity. Female participants were enlisted from May 2020 to March 2022 at the Unit of Endocrinology, Obesity Unit (Centro Italiano per la cura e il Benessere del paziente con Obesità “C.I.B.O.” and European Association for the Study of Obesity, Collaborating Centre for Obesity Management “EASO-COMs”), Clinical Medicine and Surgery Department, University of Naples Federico II; Naples, Italy.

This clinical study included a total of two outpatient visits and seven telephone interviews by a nutritionist to assess dietary adherence, any changes in physical activity levels, and the measurement of ketone bodies from capillary blood. In addition, any changes to the standard recommendations given by the nutritionist were reported. In particular, at baseline and after 45 days of the VLCKD, anthropometric measurements, HGS, and body composition were evaluated. Blood samples were obtained for the evaluation of hs-CRP.

This clinical study was previously been approved by the Federico II Ethical Committee with protocol number 50/20. All patients were informed of the study design and purpose, subsequently giving their informed consent.

### 2.2. Population Study

A total of 247 Caucasian women with a body mass index (BMI) between 30.0 and 50.9 kg/m^2^, aged 18–51 years, were consecutively enrolled in this clinical study. All these women had a history of failed dietary attempts and the desire to lose weight. An endocrinologist collected the complete medical history and ruled out any contraindications to prescribing the VLCKD, in accordance with the guidelines of the SIE [6]. Physical activity levels were assessed in all participants using a YES/NO response (at least 30 min/day aerobic exercise), as also reported in previous studies [22,23]. To increase the homogeneity of the clinical study, the exclusion criteria adopted were:

- Underweight, normal-weight, and overweight subjects;
- Male gender;
- Menopausal women, defined with at least 12 months of amenorrhea or LH levels > 30 IU/L;
- Pregnancy or breastfeeding;
- Type 1 and T2DM;
- Smokers;
- Women taking anti-inflammatory or anti-obesity drugs one month before enrollment in the study or women occasionally or currently taking medications that could influence musculoskeletal system and HGS, including anticonvulsants, steroidal and non-steroidal anti-inflammatory drugs, anti-thyroid agents, dopaminergic drugs, Parkinson’s disease drugs, or statins;
- Previous treatment with bariatric surgery;
- Clinical conditions such as immune, inflammatory, endocrine, or musculoskeletal diseases; liver or kidney failure; or neoplasms that could affect fluid balance, body composition, or HGS (ascertained after complete medical examination and appropriate laboratory tests);
- Subjects with implanted pacemakers or defibrillators (because of the potentially negative influence on the bioelectrical impedance analysis (BIA) device);
- Skin injury at the sites of electrode placement;
- Individuals unable to perform the HGS test due to conditions including osteoarticular or musculoskeletal diseases, pain, stroke, or hand injury.
- In addition, participants were evaluated at baseline, in the early follicular phase.

### 2.3. Handgrip Strength Test

Muscle strength was assessed by HGS measured using a dynamometer (Lafayette Hydraulic hand dynamometer model J00105) that provides the record of muscle strength in kilograms (kg). All measurements were performed under strictly standardized conditions by the same clinical nutritionist and the same dynamometer in order to avoid inter-observer and inter-device variability, after explaining the procedure to each participant, as previously reported [24]. Subjects were guided to obtain the correct positioning, which included sitting, with shoulders adducted and in a neutral rotation, the elbows flexed to 90°, and the forearms and wrists slightly extended (up to 30°), as recommended by the American Society of Hand Therapists [25]. Participants were asked to squeeze the dynamometer with their nondominant arm three times at maximum isometric force (for 5 s). To avoid fatigue between each repetition, 1 min of rest was observed [26]. When they were unable to perform HGS with their non-dominant hand, participants used their dominant hand. In this study, the average of the three attempts was considered for the analysis. The dynamometer was routinely checked with resistors and capacitors of known values. The cut-off point used for low HGS in women was <16 kg [20].

### 2.4. Anthropometric Measurements

As widely reported in previous studies [27,28,29], the anthropometric parameters were assessed by the same trained health care operator at a time between 8 and 10 am. The subjects, who had been fasting since the previous evening, were asked to wear light clothing and to remove their shoes during the assessments. Weight and height were used to calculate BMI (kg/m^2^). A wall stadiometer (0.5 cm approximation) and a calibrated beam scale (0.1 kg approximation) were used for height and weight measurements, respectively. World Health Organization criteria [30] were used for BMI classification into:

- Overweight (25.0–29.9 kg/m^2^);
- Obesity grade I (30.0–34.9 kg/m^2^);
- Obesity grade II (35.0–39.9 kg/m^2^);
- Obesity grade III (≥40.0 kg/m^2^).

Regarding waist circumference (WC) measurement, patients were measured while standing with feet together and arms loosely along the sides, breathing normally [22,23,27], using a nonelastic tape up to 0.1 cm at the narrowest point. In patients with the highest degree of obesity or where the narrowest waist point was not available, WC was assessed with 0.1 cm at the umbilical level using the same measuring tape [31].

### 2.5. VLCKD Intervention

Subjects who fulfilled inclusion/exclusion criteria underwent the VLCKD protocol with total meal replacement consisting of three main stages (active, re-education, and maintenance). A commercial weight-loss program was used for the VLCKD (New Penta Srl, Cuneo, Italy). The active-stage diet was planned by a skilled nutritionist and recommended by the endocrinologist. As for diet composition, total energy intake was <800 kcal/day and it was provided by 13% carbohydrates (<30 g/day), 43% protein (1.3 g/kg ideal body weight), and 44% fat. During the VLCKD, replacement meals with high biological value were used, with protein coming from whey, soy, eggs, and peas. According to international recommendations [5], supplementation of B-complex vitamins; vitamins C and E; minerals, including potassium, sodium, magnesium, and calcium; and omega-3 fatty acids at the same dosage was planned to maintain the physiological acid/base balance (PentaCal, New Penta, Ltd., Cuneo, Italy). An example of a VLCKD scheme with meal replacements is shown in Supplementary Materials File S1.

### 2.6. Bioelectrical Impedance Analysis

BIA was performed by the same skilled nutritionist using the same phase-sensitive BIA-device (an 800 A current, frequency 50 kHz BIA 101 RJL, Akern Bioresearch, Florence, Italy) [32] and under strictly standardized conditions to reduce inter-device and inter-observer variability. According to the European Society of Parenteral and Enteral Nutrition (ESPEN) guidelines [33], the assessment was performed with patients lying supine with limbs slightly apart from their body, not having indulged in any food/beverages or physical exercise in the previous six hours, nor consumed any alcohol 24 h before the assessment, as widely reported in previous studies [34,35,36]. In addition, all patients were asked to empty their bladder 30 min before the BIA evaluation, according to Kyle et al. [33]. Patients were asked to remove their shoes and socks immediately before the application of the electrodes, and the contact surfaces were cleaned with alcohol. The electrodes (BIATRODES Akern Srl; Florence, Italy) were placed on the right hand (proximal to the phalangeal-metacarpal joint on the dorsal surface) and the right foot (distal to the transverse arch on the superior surface of the right foot). The sensing electrodes were placed on the right wrist (midway between the distal projection of the radius and the ulna) and the right ankle (between the medial and lateral malleoli) [37]. Every day, the skilled nutritionist checked the device with resistors and capacitors of known values: the same observer detected an intraday variation < 2.3% for resistance (R) and <2.1% for reactance (Xc), as well as an interday variation < 3.1% for R and <2.4% for Xc. The coefficient of variation (CV) of repeated measurements of R and Xc at 50 kHz was determined in 10 females by the same observer: CVs were 1.1% for R and 1.2% for Xc, and phase angle (PhA) was derived from the conditions at 50 kHz, according to the following formula: PhA (°, degrees) = arc tangent Xc/R^x^(180/π).

### 2.7. Compliance to VLCKD

Compliance to the recommendations for the VLCKD and physical activity was assessed by weekly individual telephone counseling by an endocrinologist and nutritionist. In addition, once a week, participants were asked to measure β-hydroxybutyrate from capillary blood using test strips (Optium Xceed Blood Glucose and Ketone Monitoring System; Abbott Laboratories, Chicago, IL, USA). More specifically, at baseline and at the end of the dietary intervention (Day 45), β-hydroxybutyrate levels were measured by the nutritionist at the outpatient clinic. Furthermore, blood ketone levels were also checked directly by the patients at home once a week, in the morning on an empty stomach, preferably at the same time, and the levels were reported to the nutritionist during the telephone interview, which as carried out once a week.

### 2.8. Assay Methods

Blood samples were collected between 8.00–10.00 a.m. after an overnight fast (at least 8 h), and the samples were immediately stored at −80 °C until the assay. hs-CRP levels were assessed by a high-sensitivity nephelometric assay (CardioPhase, Siemens Healthcare Diagnostics, Marburg, Germany). The CV of intra- and interassay was <7%.

### 2.9. Statistical Analysis

Participants who completed the study, including all available examinations at both baseline and 45 days, were included in the analysis of the collected data. These were analyzed with the SPSS software package (IBM, SPSS Statistics, version 22, Armonk, NY, USA). The Kolmogorov–Smirnov test was used to test data distribution. Skewed variables were normalized by logarithm transformation and re-converted into tables and figures. The mean ± standard deviation (SD) or percentages (%) were used to present the data. The outcomes between the baseline and after the VLCKD were compared using the Student’s paired *t*-test. The chi square (χ^2^) test was used to test differences in frequency distribution across BMI, WC, HGS, and physical activity categories. Pearson’s correlation was used to assess the association between percentage changes (delta ∆%) pre/post intervention. A partial correlation was performed to adjust the associations among ∆HGS with ∆fat mass (FM) and ∆free fat mass (FFM) for confounding factors (∆BMI, ∆WC, ∆hs-CRP levels, and physical activity). Because this was a pilot study, no power calculations were performed. Therefore, all findings need to be confirmed by larger clinical trials.

## 3. Results

A total of 247 women with obesity, aged 35.4 ± 10.5 years, met the inclusion/exclusion criteria and were included in the analyses. All subjects were evaluated at baseline and after 45 days of the active stage of the VLCKD.

Adherence to the VLCKD was assessed and confirmed in all the patients by a telephone interview once a week and patients were asked about their physical activity. Additionally, each patient was called the day before the interview to instruct them to do the ketosis capillary test the next morning, while fasting and just before breakfast, and the results were reported on the day of the interview by the nutritionist. As reported in Table 2, no patient changed her physical activity levels during the 45 days of the VLCKD.

The anthropometric characteristics, body composition parameters, and hs-CRP levels of the study population, at baseline and after 45 days of active stage of the VLCKD, are shown in Table 2. Body weight, BMI, WC, and hs-CRP levels were significantly diminished (*p* < 0.001). Therefore, the distribution of subjects across the BMI categories was significantly modified, with the increase in the prevalence of overweight women (+18.6%), while the prevalence of individuals with Grade III obesity dropped to −14.2% (*p* < 0.001). Among BIA parameters, R (*p* = 0.019) and FM (*p* < 0.001) expressed as absolute value in kg were significantly reduced. In addition, FFM, expressed as absolute value in kg, showed a significant decrease (*p* = 0.001), but its percentage value was highly increased (pre 55.9 ± 6.3% vs. post 59.9 ± 6.7%; *p* < 0.0001), with an overall improvement in body composition. Interestingly, after 45 days of the VLCKD, HGS cut off was significantly improved (+11.0%, *p* < 0.001) (Table 2).

**Figure 1:**
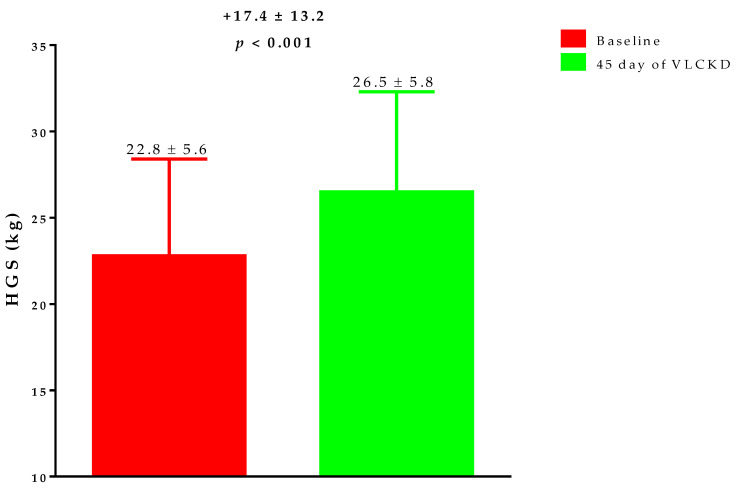
The change in HGS during the 45 days of active phase VLCKD. After 45 days of VLCKD, HGS measures were significantly increased (*p* < 0.001). VLCKD, very low-calorie ketogenic diet; HGS, handgrip strength.

Figure 1 reports the HGS changes after 45 days of active phase the VLCKD. In particular, post-VLCKD, HGS measures was significantly increased (*p* < 0.001).

The participants were grouped according to the cut off of HGS; Table 3. As shown in the table, women with HGS values higher than the cut-off point (≥16 kg) exhibited significant differences compared with those with HGS below the cut-off value (<16 kg). In particular, women with HGS values below the cut-off had higher values of BMI (*p* < 0.001), WC (*p* < 0.001), R (*p* = 0.019), FM (*p* = 0.018), hs-CRP levels (*p* = 0.017), and lower values of FFM (*p* < 0.001) than those with values above the cut-off value; Table 3.

The correlation between the changes in ∆HGS% and changes in anthropometric parameters, BIA parameters, and hs-CRP levels are reported in Table 4. Changes in ∆HGS% were significantly negatively correlated with changes in ∆BMI (*p* < 0.001), ∆WC (*p* = 0.006), ∆R (*p* < 0.001), ∆FM (*p* < 0.001), and ∆hs-CRP levels (*p* = 0.022), and positively correlated with ∆FFM (*p* < 0.001).

**Figure 2:**
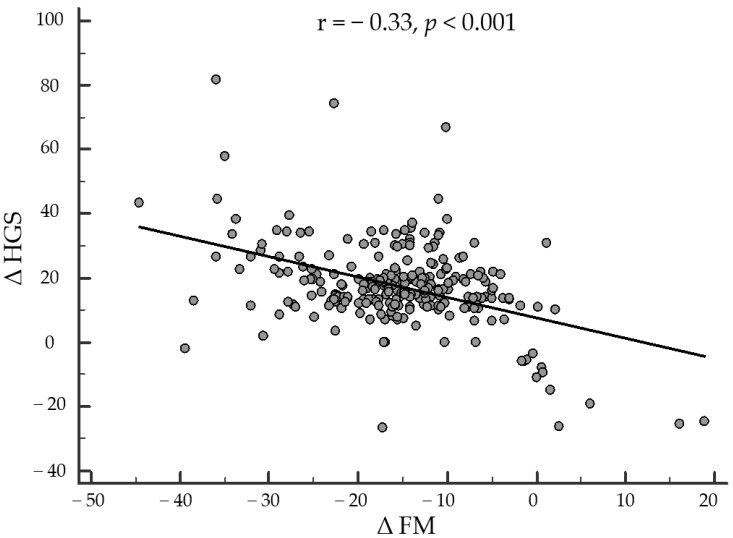
Correlation between changes in ∆HGS and ∆FM after adjusting for ∆BMI, ∆WC, ∆hs-CRP levels, and physical activity.

Finally, as reported in Figure 2, after adjusting for ∆BMI, ∆WC, ∆hs-CRP levels, and physical activity, the correlation between changes in ∆HGS and ∆FM remained statistically significant (r = −0.331, *p* < 0.001). Similarly, the correlation between ∆HGS and ∆FFM also remained statistically significant after adjustment for confounding factors (r = 0.362, *p* < 0.001). Interestingly, the correlations between ∆HGS with ∆FM and ∆FFM were also independent of the percentage of weight loss (r = −0.288, *p* < 0.001 and r = 0.395, *p* < 0.001).

After adjusting for ∆BMI, ∆WC, ∆hs-CRP levels, and physical activity, the correlation between changes in ∆HGS and ∆FM remained statistically significant (r = −0.331, *p* < 0.001). HGS, handgrip strength; FM, fat mass; BMI, body mass index; WC, waist circumference; hs-CRP, high-sensitivity C-reactive protein.

**Figure 3:**
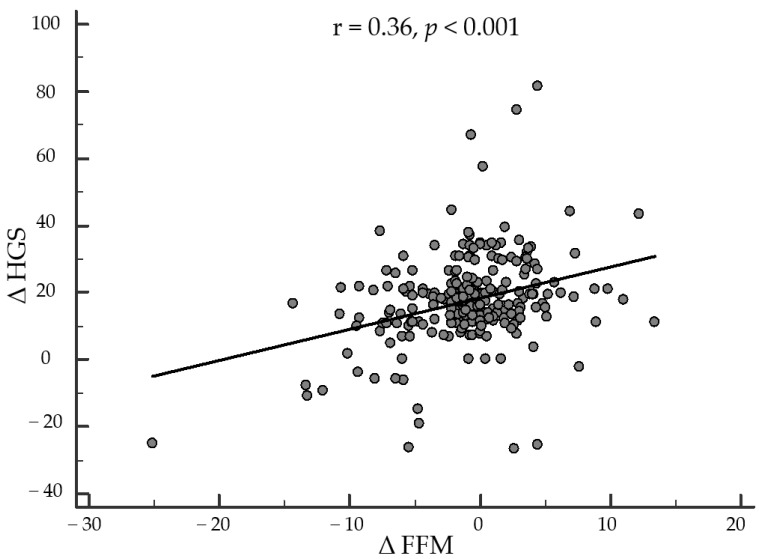
Correlation between changes in ∆HGS and ∆FFM after adjusting for ∆BMI, ∆WC, ∆hs-CRP levels, and physical activity.

After adjusting for ∆BMI, ∆WC, ∆hs-CRP levels, and physical activity, the correlation between changes in ∆HGS and ∆FFM remained statistically significant (r = 0.362, *p* < 0.001); Figure 3. HGS, handgrip strength; FFM, free fat mass; BMI, body mass index; WC, waist circumference; hs-CRP, high-sensitivity C-reactive protein.

## 4. Discussion

The aim of this prospective study was to assess the efficacy of the VLCKD in promoting changes in HGS and hs-CRP levels as a marker of inflammation in a population of women with obesity after 45 days of the active phase of the VLCKD. As expected, we obtained a significant reduction in body weight and FM in all the study participants. Of interest, FFM, expressed as an absolute value in kg, also showed a significant decrease, but its percentage value was highly increased, with an overall improvement in body composition. This finding was in line with a recent study by Paoli et al. (2021) in a group of patients with PCOS treated with a KD for 12 weeks [38]. In particular, these Authors reported that the study participants lost their weight with a significant reduction in FM accompanied by a slightly significant loss of FFM, as absolute value in kg, but with a highly significant increase in its percentage value.

As novel findings, we found that muscle strength evaluated by HGS testing increased by 17.4%, while hs-CRP levels reduced significantly. The increase in HGS was also significantly negatively correlated with changes in BMI, WC, FM, and hs-CRP levels, and positively correlated with changes in FFM. The positive association between HGS and FFM was still evident after adjusting for changes in BMI, WC, hs-CRP levels, physical activity, and percentage of weight loss.

To the best of our knowledge, this is the first study that lends support to the significant increase in FFM as percentage values during the active stage of the VLCKD, evidencing that this increase is associated either with favorable effects on muscle strength, measured by HGS testing, and the inflammatory status evaluated by hs-CRP levels. In addition, we characterized the study participants according to the results of the HGS test. Of interest, the study participants with HGS values lower than the cut-off point also exhibited higher BMI, visceral adiposity measured by WC, FM, and hs-CRP levels, as well as lower values of FFM compared with their counterpart with HGS values above the cut-off point. Increasing the knowledge regarding the possible mechanism involved in the preservation of FFM is a crucial factor in the maintenance of long-term weight loss after completing the VLCKD protocols.

Paoli et al. (2019) extensively evaluated the relationship between KD and muscle mass regulatory pathways, and suggested that a protective role for muscle anabolism could be mediated by the activation of the AMP-activated protein kinase, with changes in the Akt/mTOR pathways [12]. In addition, the VLCKD has been demonstrated to reduce inflammation more significantly than other dietary regimens [39]. The anti-inflammatory effects of KD were recently reported in an extensive review of KD in the management of sarcopenic obesity [40]. In particular, a recently published extensive review reported the beneficial effects of ketone bodies in reducing inflammation and metabolic complications involved in NAFLD pathogenesis [41]. Among ketone bodies, β-hydroxybutyrate has been shown to exert both anti-inflammatory and anti-catabolic effects on human skeletal muscle by inhibiting the activation of the Nf-κB pathway [2,42]. Very recently, Camajani et al. [43] described the beneficial effects of the VLCKD combined with physical exercise in reducing FM, improving metabolic profile, and preserving skeletal muscle performance in a woman with sarcopenic obesity two weeks after hospitalization for severe COVID-19 disease. In the context of a link between the inflammatory status and FFM, a very recently published study reported the benefits of dietary n-3 polyunsaturated fatty acids for muscle function in healthy older adults to target inflammation and to improve the “anabolic resistance” to exercise associated with aging [44], while an extensive review examined the anti-inflammatory and antioxidative properties of extra virgin olive oil in delaying/preventing loss of muscle mass and function [45].

Of note, in our study group, the positive association between FFM and HGS appears to be independent regarding changes in hs-CRP levels. In line with the current literature [46], this observation pointed out the importance of ensuring an adequate intake of protein, both in terms of quantity (1.2–1.5 g/kg ideal body weight), and quality, with high-biological-value protein preparations, such as those we used in this study. In this context, the results of a very recent meta-analysis on the use of protein intake to support muscle mass and function in healthy adults showed that there was not a significant difference when performing the analysis of subgroups by age [47]. However, it is tempting to speculate that the low insulin-to-glucagon ratio induced by the severe restriction in carbohydrates intake during the active phase of the VLCKD could be associated with multiple changes in endocrine axes involved in the regulation of body composition [38,48,49,50], including the somatotropic axis [51].

We are aware that our study has some limitations, but also a number of strengths. First, despite the fact that this pilot study was conducted on a large study population considering the VLCKD setting, the study involved only a single-center, and this may have introduced some selection bias. However, the homogeneity of the population was enhanced by including only premenopausal women to avoid the possible influence of estrogenic decline on changes in muscle strength and mass.

Second, we did not include a control group on a non-KD. Therefore, we can only speculate that ketosis, rather than weight loss per se, could exert the favorable changes in the HGS. Of note, these changes were also independent of physical activity, as the participants in this study did not change their physical activity during the 45 days of the VLCKD. Nevertheless, the beneficial effects of brief VLCKD on inflammation in patients with overweight and obesity have been extensively reported [1,2,7,11,42], along with the superiority of the VLCKDs over other low-calorie dietary regimens for inducing weight loss [52].

Third, we have not assessed other inflammatory and antioxidant markers. However, CRP represents the most investigated inflammatory biomarker for several diseases [53]. Therefore, we cannot exclude the possibility that, besides the intervention of the VLCKD, other factors could also play an important role in the variations in HGS values. Finally, being a pilot study, we did not extend our observations to subsequent stages of the VLCKD to confirm the association between FFM and HGS. However, the very short observation length prevented patients from abandoning the protocol.

## 5. Conclusions

In this pilot study, we first reported that, along with a significant reduction in body weight and an overall improvement in body composition and inflammatory status, the muscle strength evaluated by HGS testing increased in a population of women with obesity after 45 days of the active stage of the VLCKD, even following adjustments for common confounding variables. These findings also lend support to the increase in FFM during the active stage of the VLCKD and show the usefulness of the HGS test in the nutritional management of patients with obesity as a possible additional diagnostic tool to CRP to evaluate changes in the inflammation status and the correct planning of the VLCKD protocol stages. However, further studies extending the observations to subsequent stages of the VLCKD compared with other hypocaloric dietary programs to unravel the relative role of ketosis and weight loss, are mandatory to confirm the association between FFM and HGS.

## Figures and Tables

**Table 1:** Strong and weak indications and contraindications to VLCKD of SIE.

VERY LOW-CALORIE KETOGENIC DIETS (VLCKDS)	
STRONG RECOMMENDATIONS	CONTRAINDICATIONS
Severe obesity	Pregnancy and breastfeeding kidney failure
Severe obesity before bariatric surgery	Moderate-to-severe chronic kidney disease
Sarcopenic obesity	Liver failure
Obesity associated with: T2DM (preserved beta cell function) Hypertriglyceridemia Hypertension	Rare disorders: porphyria, carnitine deficiency, carnitine palmitoyltransferase deficiency, carnitine-acylcarnitine translocase deficiency, mitochondrial fatty acid β-oxidation disorders, and pyruvate carboxylase deficiency
Pediatric obesity associated with epilepsy and/or with a high level of insulin resistance and/or comorbidities, not responsive to standardized diet	Respiratory failure unstable angina, hearth failure (NYHA III–IV), recent stroke or myocardial infarction (<12 months), and cardiac arrhythmias
**WEAK RECOMMENDATIONS** Obesity associated with dysbiosis; Obesity associated with dyslipidemia; Obesity associated with NAFLD; Obesity associated with heart failure (NYHA I–II); Obesity associated with atherosclerosis; Male obesity secondary hypogonadism; Obesity associated with PCOS; Menopausal transition-related obesity; Neurodegenerative disorders.	Eating disorders and other severe mental illnesses, alcohol, and substance abuse
	Type 1 diabetes mellitus, latent autoimmune diabetes in adults, β-cell failure in T2DM, use of SGLT2 inhibitors
	Active/severe infections and frail elderly patients,
	48 h prior to elective surgery or invasive procedures and perioperative period

SIE, Italian Society of Endocrinology; T2DM, type 2 diabetes mellitus; NAFLD, non-alcoholic fatty liver disease; NYHA, New York Heart Association; PCOS, polycystic ovary syndrome; SGLT, sodium-dependent glucose cotransporters.

**Table 2:** Anthropometric measurements, HGS, physical activity, body composition, and inflammatory biomarker of the study population at baseline and after 45 days of the active stage of VLCKD.

Parameters	Participant Baseline Mean ± SD or Number (%) *n* = 247	Participant 45th Day of VLCKD Mean ± SD or Number (%) *n* = 247	∆%	* p-Value
**Weight (kg)**	99.4 ± 15.2	91.9 ± 14.3	−7.5 ± 3.1	**<0.001**
**BMI (kg/m^2^)**	37.3 ± 4.5	34.5 ± 4.3		**<0.001**
Overweight (*n*, %)	-	46, 18.6%	18.6%	χ^2^ = 48.54, ***p* < 0.001**
Grade I obesity (*n*, %)	88, 35.6%	94, 38.1%	2.5%	χ^2^ = 0.22, *p* = 0.641
Grade II obesity (*n*, %)	94, 38.1%	77, 31.2%	−6.9%	χ^2^ = 2.29, *p* = 0.130
Grade III obesity (*n*, %)	65, 26.3%	30, 12.1%	−14.2%	χ^2^ = 15.07, ***p* < 0.001**
**WC (cm)**	108.0 ± 14.5	101.1 ± 13.8	−6.3 ± 5.0	**<0.001**
WC < 88 cm	18, 7.3%	45, 18.2%	−10.9%	χ^2^ = 12.30, ***p* < 0.001**
WC ≥ 88 cm	229, 92.7%	202, 81.8%		
**HGS (kg)**				
<16 kg	36, 14.6%	9, 3.6%	+11.0%	χ^2^ = 16.53, ***p* < 0.001**
≥16 kg	211, 85.4%	238, 96.4%		
**Physical Activity**				
Yes	78, 31.6%	78, 31.6%	0%	χ^2^ = 0.00, *p* = 0.922
No	169, 68.4%	169, 68.4%		
**BIA parameters**				
R (Ω)	468.8 ± 70.5	472.8 ± 64.4	+1.4 ± 9.5	0.129
Xc (Ω)	45.7 ± 9.5	49.8 ± 9.8	10.3 ± 16.2	**<0.001**
FM (kg)	44.5 ± 12.5	37.6 ± 11.5	−15.6 ± 9.0	**<0.001**
FFM (kg)	54.9 ± 5.7	54.4 ± 5.7	−0.9 ± 4.5	**0.001**
**hs-CRP levels (mg/L)**	3.8 ± 4.3	1.9 ± 2.6	−39.9 ± 44.6	**<0.001**

* A *p* value in bold type denotes a significant difference (*p* < 0.05). SD, standard deviation; VLCKD, very low-calorie ketogenic diet; BMI, body mass index; WC, waist circumference; HGS, handgrip strength; R, resistance; Xc, reactance; FM, fat mass, FFM, free fat mass; hs-CRP, high-sensitivity C-reactive protein.

**Table 3:** Anthropometric measurements, body composition, and hs-CRP levels of the study population baseline grouped according to the cut-off of HGS.

	Participant Baseline *n* = 247		
Parameters	HGS <16 kg Mean ± SD *n* = 36	HGS ≥16 kg Mean ± SD *n* = 211	* p-Value
**Age (years)**	36.7 ± 9.1	35.2 ± 10.7	0.424
**Weight (kg)**	111.0 ± 17.9	97.4 ± 13.7	**<0.001**
**BMI (kg/m^2^)**	43.1 ± 3.7	36.3 ± 3.8	**<0.001**
**WC (cm)**	119.1 ± 12.4	106.1 ± 14.0	**<0.001**
**BIA parameters**			
R (Ω)	494.2 ± 85.9	464.4 ± 66.8	**0.019**
Xc (Ω)	43.4 ± 8.9	46.1 ± 9.5	0.111
FM (kg)	57.9 ± 14.8	42.2 ± 10.5	**0.018**
FFM (kg)	53.1 ± 4.6	55.2 ± 5.8	**<0.001**
**hs-CRP levels (mg/L)**	6.4 ± 6.8	3.3 ± 3.2	**0.017**

* A *p* value in bold type denotes a significant difference (*p* < 0.05). HGS, handgrip strength; SD, standard deviation, BMI, body mass index; WC, waist circumference; R, resistance; Xc, reactance; FM, fat mass, FFM, free fat mass; hs-CRP, high-sensitivity C-reactive protein.

**Table 4:** Correlation among ∆%HGS with age, ∆%anthropometric parameters, ∆% body composition parameters, and ∆% of hs-CRP levels.

Parameters	R	* p-Value
Age	−0.068	0.290
**∆**BMI (%)	−0.356	**<0.001**
**∆**WC (%)	−0.176	**0.006**
**∆**R (%)	−0.334	**<0.001**
**∆**Xc (%)	0.051	0.425
**∆**FM (%)	−0.432	**<0.001**
**∆**FFM (%)	0.320	**<0.001**
**∆**hs-CRP levels (%)	−0.171	**0.022**

* A *p* value in bold type denotes a significant difference (*p* < 0.05). BMI, body mass index; WC, waist circumference; R, resistance; Xc, reactance; FM, fat mass, FFM, free fat mass; hs-CRP, high-sensitivity C-reactive protein.

## Data Availability

The data presented in this study are available on request from the corresponding author.

## Supplementary Materials

The following supporting information can be downloaded at: https://www.mdpi.com/article/10.3390/nu14194213/s1, Table S1: Example of VLCKD diet therapy with meal replacement (New Penta Srl, Cuneo, Italy).

## Abbreviations

VLCKDs	very low-calorie ketogenic diets
EASO	European Association for the Study of Obesity
SIE	Italian Society of Endocrinology
T2DM	type 2 diabetes mellitus
NAFLD	non-alcoholic fatty liver disease
NYHA	New York Heart Association
PCOS	polycystic ovary syndrome
SGLT	sodium-dependent glucose cotransporters
KDs	ketogenic diets
HGS	handgrip strength
hs-CRP	high-sensitivity C-reactive protein
BMI	body mass index
BIA	bioelectrical impedance analysis
WC	waist circumference
ESPEN	European Society of Parenteral and Enteral Nutrition
R	resistance
Xc	reactance
CV	coefficient of variation
PhA	phase angle
SD	standard deviation
FM	fat mass
FFM	free fat mass
