# Supplementary material for: Impact of a Very Low-Calorie Ketogenic Diet (VLCKD) on Changes in Handgrip Strength in Women with Obesity
Source: Nutrients. 2022 Oct 10;14(19):4213. doi: 10.3390/nu14194213 (PMC9571084; doi:10.3390/nu14194213)
Supplement: Supplementary file 1 [file nutrients-14-04213-s001.zip › nutrients-1913627-supplementary.pdf]

TABLE S1. EXAMPLE OF VLCKD DIET THERAPY WITH MEAL REPLACEMENT

(New Penta Srl, Cuneo, Italy)

Breakfast (meal replacement, cappuccino drink)*

AVERAGE NUTRITIONAL ANALYSIS	
Kcal/KJ	91 / 386
Fat (g)	0,5
of which saturated fatty acids (g)	0,4
Carbohydrates (g)	2,7
of which sugars (g)	1,7
Fiber (g)	1,7
Protein (g)	18
Salt (g)	0,33

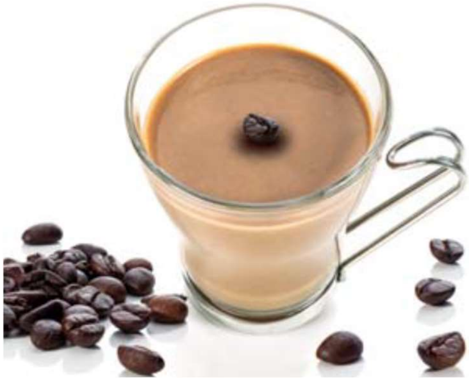

Mid-morning snack (meal replacement, milkshake-flavored drinks)*

AVERAGE NUTRITIONAL ANALYSIS	
Kcal/KJ	88 / 371
Fat (g)	0,7
of which saturated fatty acids (g)	0,2
Carbohydrates (g)	1,4
of which sugars (g)	1,1
Fiber (g)	2,5
Protein (g)	18
Salt (g)	0,32

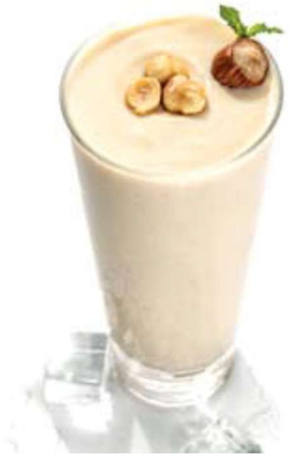

Lunch, meal replacement (protein pasta)*

AVERAGE NUTRITIONAL ANALYSIS	
Kcal/KJ	143 / 605
Fat (g)	1,3
of which saturated fatty acids (g)	0,9
Carbohydrates (g)	1,6
of which sugars (g)	1,2
Fiber (g)	3,2
Protein (g)	15
Salt (g)	0,27

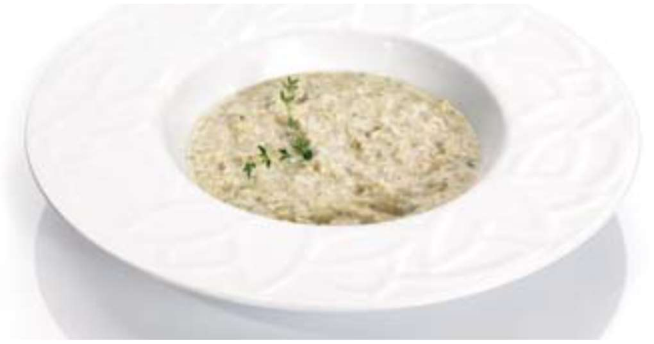

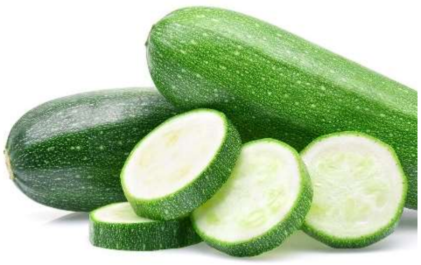

+ 200 g of low glycaemic index vegetables (e.g. courgettes)

+ 15 g of extra virgin olive oil

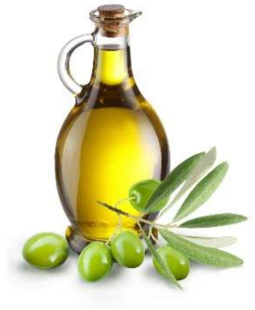

Dinner, meal replacement (type omelette)*

AVERAGE NUTRITIONAL ANALYSIS	
Kcal/KJ	113 / 476
Fat (g)	2,6
of which saturated fatty acids (g)	0,1
Carbohydrates (g)	2,5
of which sugars (g)	0,5
Fiber (g)	0,5
Protein (g)	20
Salt (g)	1,4

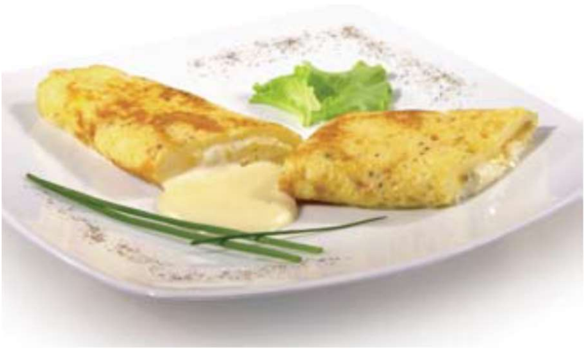

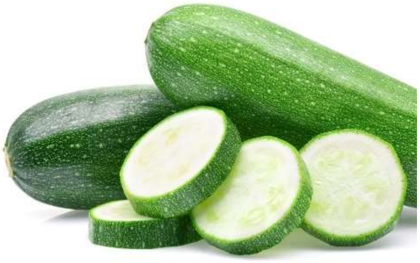

+ 200 g of low glycaemic index vegetables (e.g. courgettes)

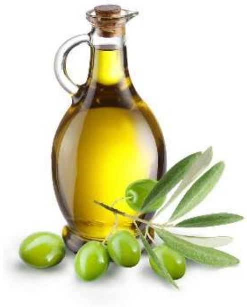

+ 15 g of extra virgin olive oil

In addition:

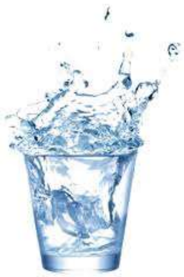

+ Drink at least 2.5 liters of water *per* day

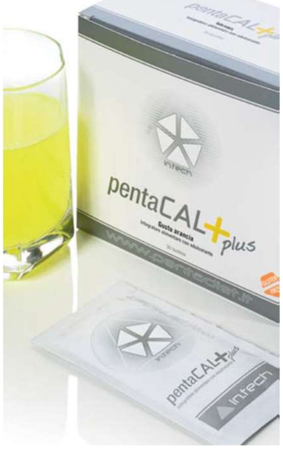

+ Multi-vitamin and saline supplements to maintain physiological ac-id/base balance (PentaCal, New Penta, Srl., Cuneo, Italy) (B complex vitamins, C and E vitamins, minerals, including potassium, sodium, magnesium, calcium, and omega-3 fatty acids.

Calories and nutrients from meal replacements	
Kcal/KJ	435/1838
Fat (g)	5,1
of which saturated fatty acids (g)	1,6
Carbohydrates (g)	8,2
of which sugars (g)	4,5
Fiber (g)	7,9
Protein (g)	71
Salt (g)	2,32

Calories and nutrients from EVOO and vegetables		
	EVOO (30 g)	Vegetables (e.g. courgettes) (200 g + 200 g)
Kcal/KJ	269,7/1108,8	44/192
Fat (g)	29,97	0,4
of which saturated fatty acids (g)	0	0
Carbohydrates (g)	0	5,6
of which sugars (g)	0	0
Fiber (g)	0	5,2
Protein (g)	0	5,2
Salt (g)	0	4

Average values of the VLCKD	
**Kcal/KJ**	748,7/3138,8
**Fat (g)**	35,47
of which saturated fatty acids (g)	1,6
**Carbohydrates (g)**	13,8
of which sugars (g)	4,5
**Fiber (g)**	7,9
**Protein (g)**	76,2
**Salt (g)**	6,32

*** https://pentadiet.it/prodotti**
